# Supplementary material for: Complement activation drives the phagocytosis of necrotic cell debris and resolution of liver injury
Source: Front Immunol. 2024 Dec 17;15:1512470. doi: 10.3389/fimmu.2024.1512470 (PMC11696981; doi:10.3389/fimmu.2024.1512470)
Supplement: Supplementary file 1 [file DataSheet1.docx]

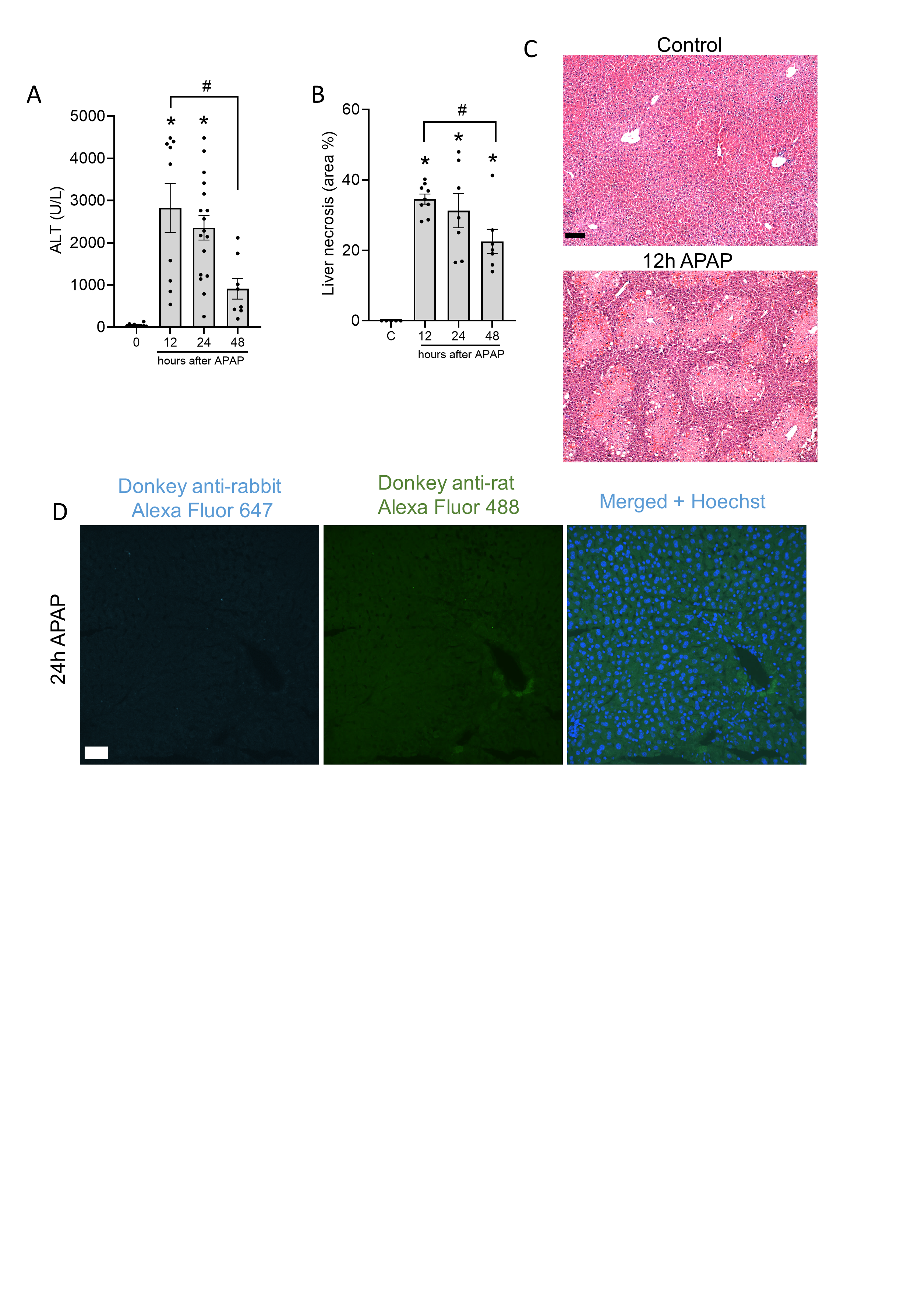


**Supplemental Figure 1. Liver injury in response to paracetamol (APAP) overdose in mice.** (A) Serum ALT levels in mice 12, 24 and 48h after receiving an overdose of 600 mg/kg APAP. (B) Area of necrosis in the liver of mice 12, 24 and 48h after receiving an overdose of 600 mg/kg APAP, determined by histopathology. (C) Representative H&E staining images of a control liver and necrotic liver 12h after APAP administration. Scale bar represents 100 µm. Image quantifications were pooled from 10 fields of view. Images were taken using a BX41 optical microscope (Olympus) and obtained using the Moticam 2500 camera (Motic) and Motic Image Plus 2.0ML software. (D) Cryosections of WT mice 24h after APAP overdose and stained with secondary antibodies only. Hoechst was used to stain the nuclei (blue). Scale bar represents 50 µm. Data are represented as mean ± SEM. Each dot represents a single mouse. At least 4 mice were used per group. *p≤0.05 compared to control; #p≤0.05 between indicated groups. APAP=acetaminophen, ALT=alanine aminotransferase.


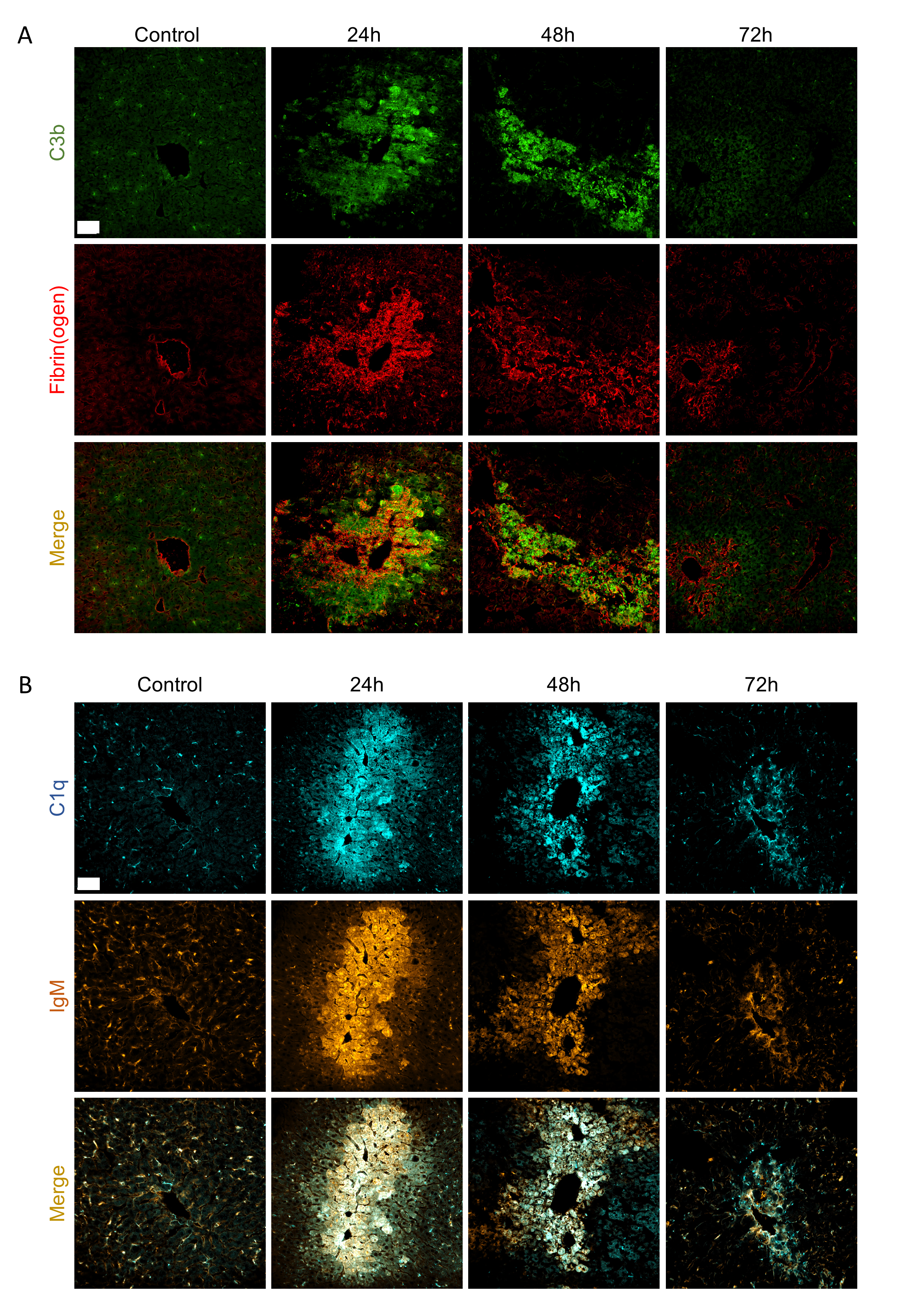


**Supplemental Figure 2. Time-response evaluation of C1q and C3b deposition at the sites of necrotic injury in the liver.** (A,B) Representative immunofluorescence images of liver cryosections from control mice and mice 24, 48 and 72 hours after receiving an overdose of acetaminophen (APAP; 600 mg/kg). Green: (i)C3b, red: fibrin(ogen), Orange: IgM; Cyan: C1q. Scale bar represents 50 µm. Images were captured using a Zeiss Axiovert 200M fluorescence microscope with a 20X objective.


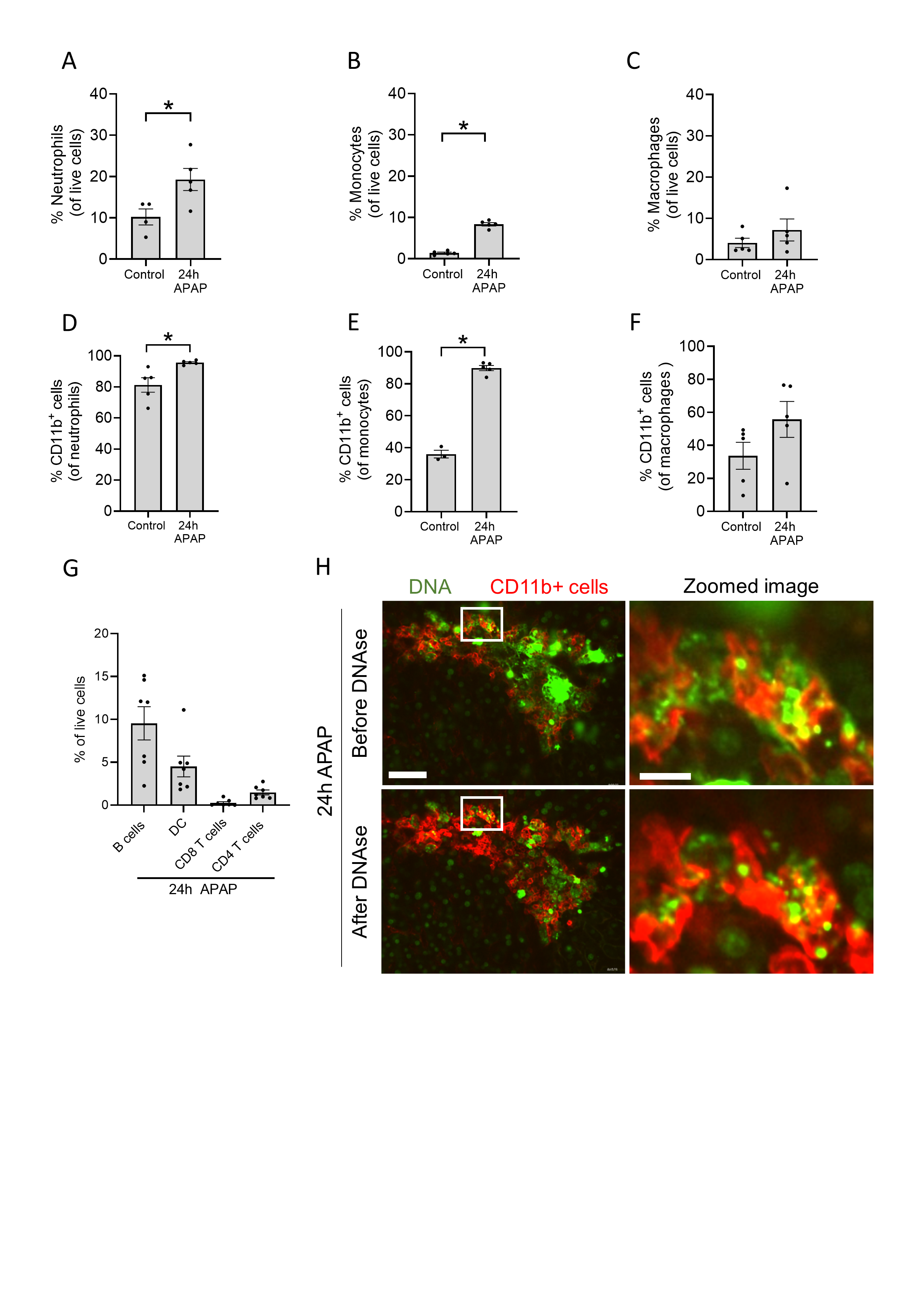


**Supplemental figure 3. DNAse treatment validates the internalization of DNA debris in CD11b+ leukocytes.** (A-F) Flow cytometry of liver non-parenchymal cells identifying the percentage of neutrophils (Ly6G^+^), monocytes (Ly6G^-^ / Ly6C^+^ / CCR2^+^) and macrophages (F4/80^+^) and the percentage of cells expressing CD11b in the injured liver 24h after APAP overdose. (G) Flow cytometry of liver non-parenchymal cells of mice receiving an APAP overdose 24h prior, identifying the percentage of B cells (CD3^-^/CD19^+^), CD4^+^ T cells (CD19^-^/CD3^+^/CD4^+^), CD8^+^ T cells (CD19^-^/CD3^+^/CD8^+^) and dendritic cells (CD11c^+^). (H) Representative IVM images of the injured liver 24h after an APAP overdose. 2 µl of the cell-impermeable DNA dye Sytox Green was injected 1h before imaging. Leukocytes were labeled with anti-CD11b antibody (red). 1 mg DNAse I was injected intravenously to remove extracellular DNA. Scale bar represents 50 µm, scale bar zoomed image represents 10 µm. Images were captured using an Andor Dragonfly High-Speed Confocal Microscope, with a 25X objective. Data are represented as mean ± SEM. Each dot represents a single mouse. At least 3 mice were used per group. *p≤0.05 compared to control.


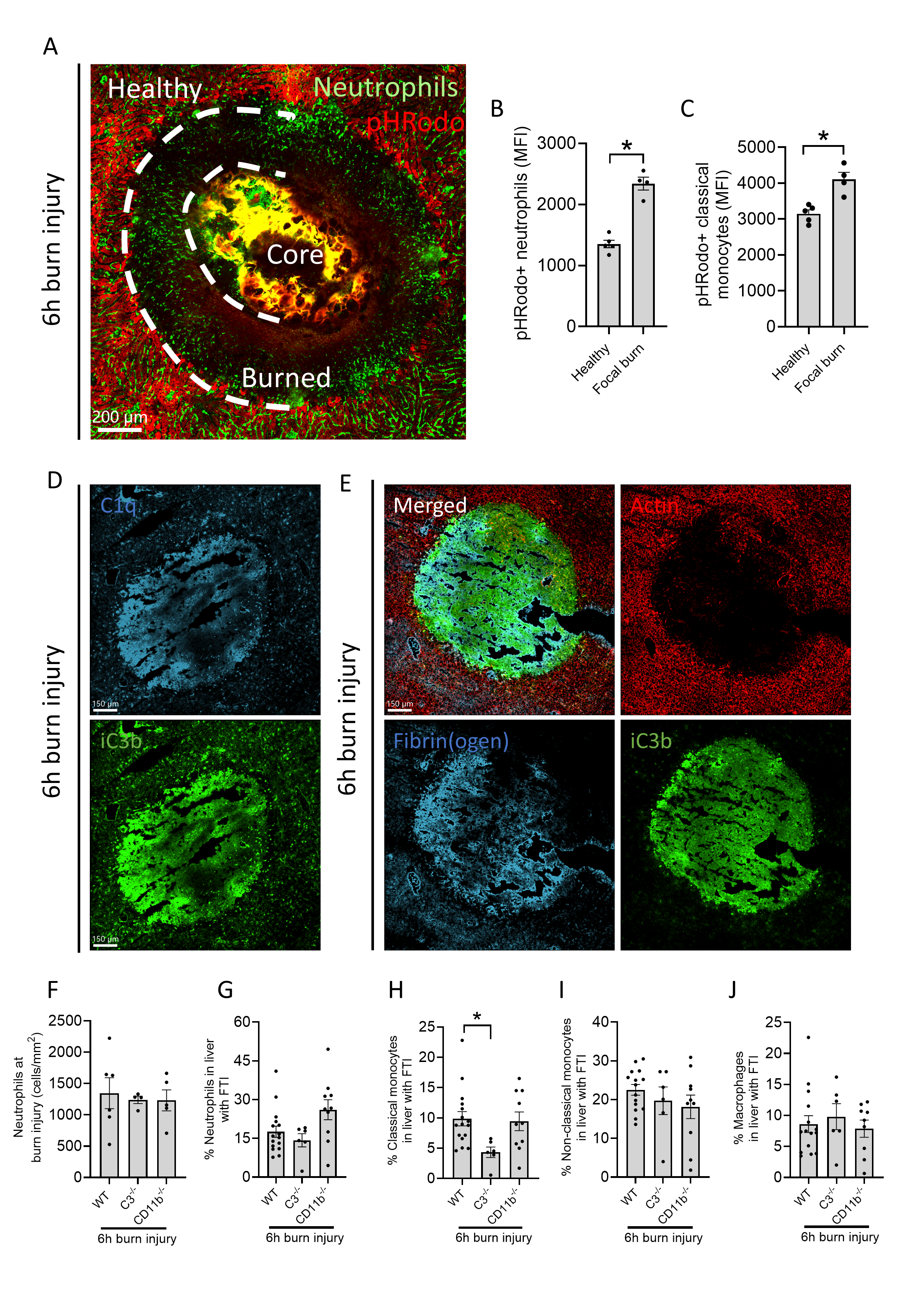


**Supplemental figure 4. Quantification and visualization of leukocytes and complement proteins in the focal thermal injury of the liver.** (A) Representative image of a 6h burn injury showing the different injury zones (core of injury, burned and healthy areas). Green: neutrophils (Ly6G); Red, pHRodo debris. Scale bar represents 200 µm. (B-C) Mean fluorescence intensity of pHRodo-labeled necrotic debris in neutrophils and classical monocytes in healthy and burned areas. (D) Representative immunofluorescence images of liver cryosections from 6h burn injury. Green: (i)-C3b; Cyan: C1q. Scale bar represents 150 µm.
(E) Representative immunofluorescence images of liver cryosections from 6h burn injury. Green: (i)-C3b; Cyan: Fibrin(ogen); Red: f-actin. Scale bar represents 150 µm. (F) Number of neutrophils per mm^2^ of burn injury. Neutrophils were manually counted from IVM images of burn injury sites. (G-J) Flow cytometry of liver non-parenchymal cells identifying neutrophils (Ly6G^+^), classical monocytes (Ly6C^+^ / CX_3_CR1^-^ / CCR2^+^), non-classical monocytes (Ly6C^+^ / CX_3_CR1^-^ / CCR2^+^) and macrophages (F4/80^+^) 6h after burn in WT, C3^-/-^ and CD11b^-/-^ livers. FTI=focal thermal injury. Data are represented as mean ± SEM. Each dot represents a mouse. At least 4 mice were used per group.*p≤0.05


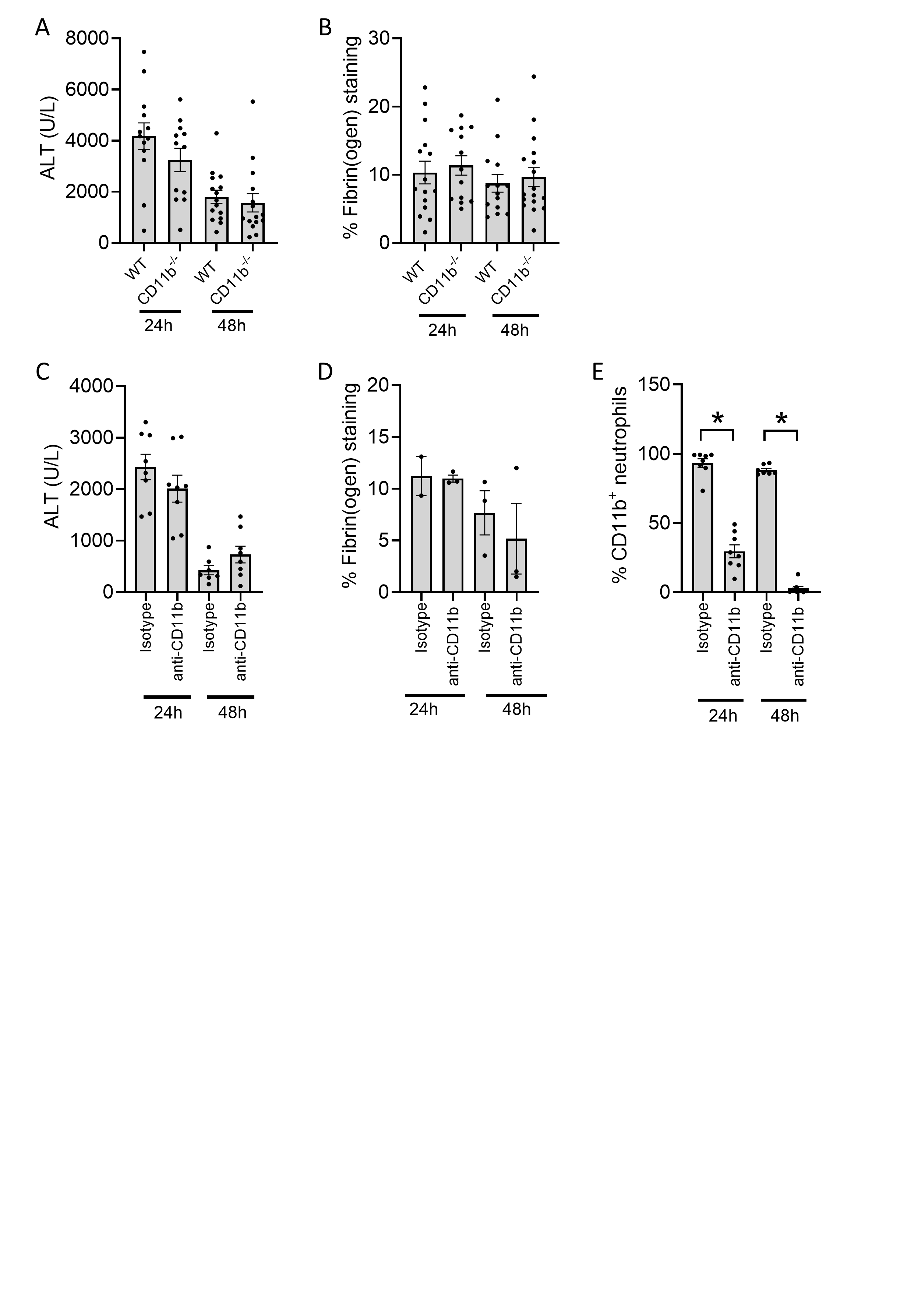


**Supplemental figure 5. CD11b does not participate in the recovery from drug-induced liver injury.** (A) ALT levels of WT and CD11b^-/-^ mice challenged with APAP for 24 or 48h. (B) Quantification of the fibrin(ogen)^+^ area fraction in liver cryosections of WT and CD11b^-/-^ mice challenged with APAP in experiment (A). (C) ALT levels of mice challenged with APAP, treated with 40 µg isotype control or 40 µg anti-CD11b blocking antibody 6 and 12h post-APAP. Samples were collected at either 24 or 48h post APAP overdose. (D) Quantification of the fibrin(ogen)^+^ area fraction in liver cryosections of experiment (C). (E) Percentage of neutrophils expressing CD11b determined by flow cytometry of experiment (C). Data are represented as mean ± SEM. Each dot represents a mouse. At least 2 mice were used per group.**p≤0.05.


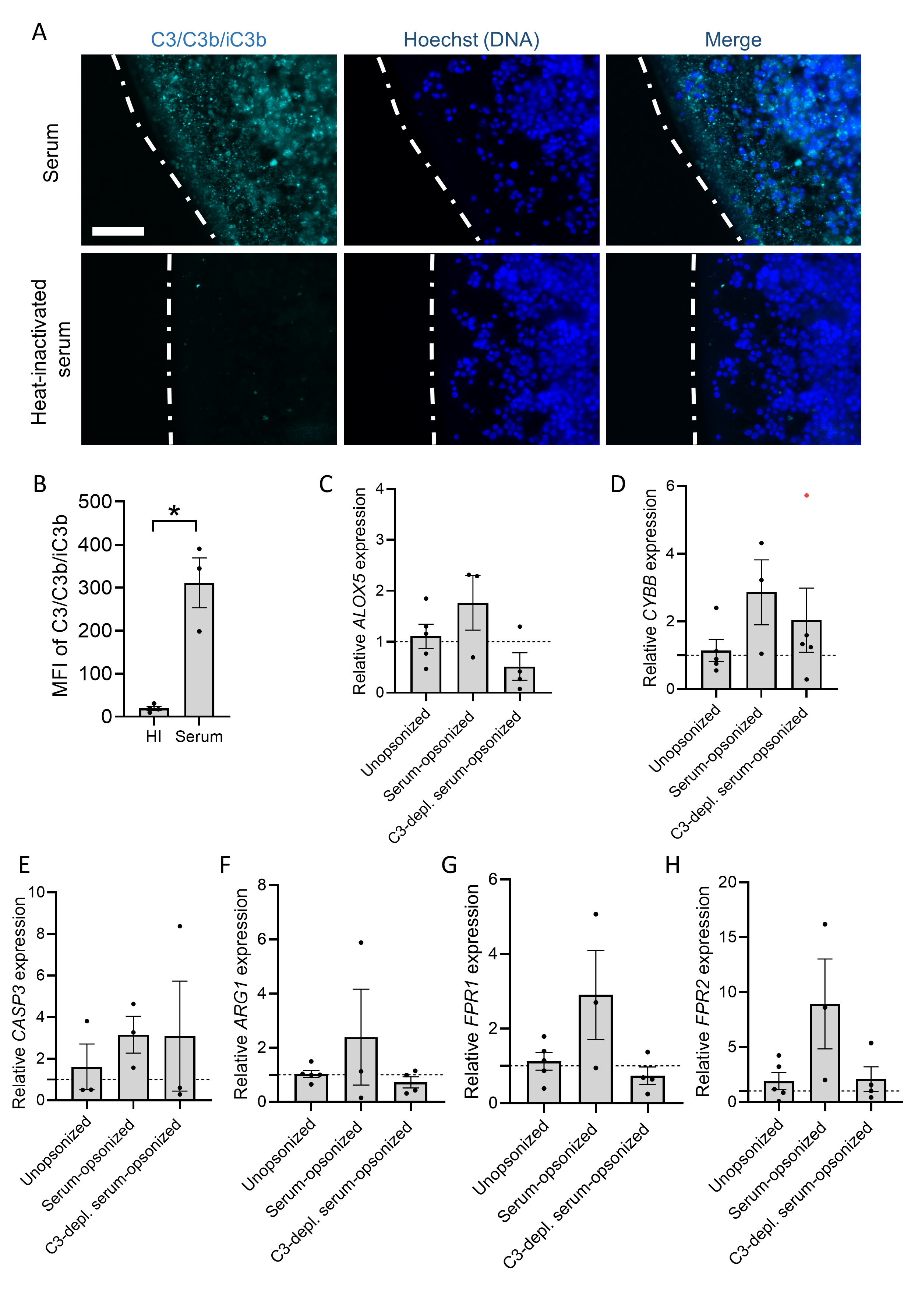


**Supplemental figure 6. Complement activation on purified necrotic debris *in vitro.*** (A) Representative images of necrotic debris form HepG2 cells opsonized with 20% serum or heat-inactivated serum. DNA debris is labeled with Hoechst (blue) and C3/C3b/iC3b in Cyan. Scale bar represent 100 µm. (B) Mean fluorescence intensity of C3/C3b/iC3b labeling on in vitro necrotic debris opsonized with 20% serum or heat-inactivated (HI) serum. (C-H) Gene expression of human neutrophils incubated with unopsonized, serum-opsonized or C3-depleted serum-opsonized human necrotic debris. Data is normalized to the average expression of 3 housekeeping genes (GAPDH, 18s and CDKN1A) and represented as 2^–∆∆Ct^ relative to the unopsonized group. Images were taken with a Zeiss Axiovert 200M fluorescence microscope and analyzed with FIJI. Data are represented as mean ± SEM. Significant outliers are indicated as red dots.*p≤0.05.
